# Phage-host interactions in *Streptococcus thermophilus*: Genome analysis of phages isolated in Uruguay and ectopic spacer acquisition in CRISPR array

**DOI:** 10.1038/srep43438

**Published:** 2017-03-06

**Authors:** Rodrigo Achigar, Alfonso H. Magadán, Denise M. Tremblay, María Julia Pianzzola, Sylvain Moineau

**Affiliations:** 1 Laboratorio de Microbiología Molecular, Departamento de Biociencias, Facultad de Química, Universidad de la República, Montevideo, Uruguay; 2 Département de Biochimie, de Microbiologie et de Bioinformatique & PROTEO, Faculté des Sciences et de Génie, Félix d’Hérelle Reference Center for Bacterial Viruses & GREB, Faculté de Médecine Dentaire, Université Laval, Québec City, Québec, G1V 0A6, Canada

## Abstract

Three *cos*-type virulent *Streptococcus thermophilus* phages were isolated from failed mozzarella production in Uruguay. Genome analyses showed that these phages are similar to those isolated elsewhere around the world. The CRISPR1 and CRISPR3 arrays of the three *S. thermophilus* host strains from Uruguay were also characterized and similarities were noted with previously described model strains SMQ-301, LMD-9 and DGCC7710. Spontaneous bacteriophage-insensitive *S. thermophilus* mutants (BIMs) were obtained after challenging the phage-sensitive wild-type strain Uy02 with the phage 128 and their CRISPR content was analyzed. Analysis of 23 BIMs indicated that all of them had acquired at least one new spacer in their CRISPR1 array. While 14 BIMs had acquired spacer at the 5′-end of the array, 9 other BIMs acquired a spacer within the array. Comparison of the leader sequence in strains Uy02 and DGCC7710 showed a nucleotide deletion at position -1 in Uy02, which may be responsible for the observed ectopic spacer acquisition. Analysis of the spacer sequences upstream the newly acquired ectopic spacer indicated presence of a conserved adenine residue at position -2. This study indicates that natural strains of *S. thermophilus* can also acquire spacers within a CRISPR array.

*Streptococcus thermophilus* is widely used for large-scale manufacture of a variety of cheeses and yogurts^1^. It is considered the second most important dairy species after *Lactococcus lactis*^2^. However, phage attacks are still today a significant risk to industrial milk fermentations driven by *S. thermophilus*^3^,^4^. Virulent phage contaminations can result in the lysis of the added starter cultures in the fermentation vat, causing slow fermentation, low quality products or in worst cases, total process failure^3^.

All currently known phages of *S. thermophilus* belong to the *Siphoviridae* family of the *Caudovirales* order^5^. They are characterized by isometric capsids (60 nm) and long, non-contractile tails (220 to 330 nm) as well as a double-stranded DNA genome^5^. *S. thermophilus* phages were originally classified in only two groups based on their distinctive DNA packaging mechanism (*cos* or *pac*) and their structural protein profiles^6^. Comparative genome analyses, particularly of the morphogenesis module, confirmed this previous classification^6^. Two other *S. thermophilus* phages groups, albeit rare, were recently identified^7^,^8^. Despite this expanding number of groups, *S. thermophilus* phage genomes are similarly organized into modular regions and the genes coding for DNA replication and host lysis are highly conserved. Bioinformatic analyses have led to a better assessment of their origins, relationships with other phages, and mechanisms responsible for their diversity^1^. The latter is mostly driven by point mutations and recombination^1^.

*S. thermophilus* phages are more often isolated from cheese plants and they show greater genomic diversity than those isolated from yogurt facilities^9^. The preferred explanation for this observation is the differences of the fermentation conditions. A significant volume of liquid whey and open vats in cheese industry favor the dispersion of phages^9^. Phage diversity may also be due to the rotation of several distinct starter cultures in cheese manufacturing.

In Uruguay, the dairy industry has sustained significant growth in the last decade. It is well documented that expanded productivity within existing production facilities may lead to fermentation failures due to virulent phages^10^,^11^. Therefore, strategies to control phages are mandatory to minimize their negative impact on dairy production^12^,^13^. One of the key tactics to limit phage propagation is the use of phage-resistant cultures. *S. thermophilus* appears to rely heavily on its CRISPR-Cas systems to protect itself against phages. Clustered regularly interspaced short palindromic repeats (CRISPR) and their associated *cas* genes are key components of an adaptive phage defense system^14^,^15^. Briefly, using various Cas proteins^15^, bacteria can acquire resistance to phages by integrating short fragments (spacers) of invading nucleic acids from phages, including defective phages^16^ at the leader end (5′-end) of the CRISPR array^17^. Then, the CRISPR is transcribed and processed into small interfering RNAs (crRNAs)^18^,^19^. The final step of the mechanism is called interference, where crRNA-Cas ribonucleoprotein complexes mediate base-pairing target recognition and specific cleavage of phage DNA in subsequent phage infections^20^,^21^. Overall, *S. thermophilus* strains have been shown to have up to four CRISPR-Cas systems (CR1– CR4)^15^. However, most *S. thermophilus* strains possess two active (for spacer acquisition and interference) type II-A CRISPR-Cas systems (CR1 and CR3)^16^,^19^.

Here, we report the complete genomic sequence of three Uruguayan virulent *S. thermophilus* phages, isolated from failed mozzarella cheese fermentations, and the CRISPR content analysis of their three respective *S. thermophilus* hosts. We also generated and characterized spontaneous *S. thermophilus* bacteriophage-insensitive mutants and analyzed their CRISPR array.

## Results

### Characterization of three virulent *S. thermophilus* phages from Uruguay

**Figure 1:**
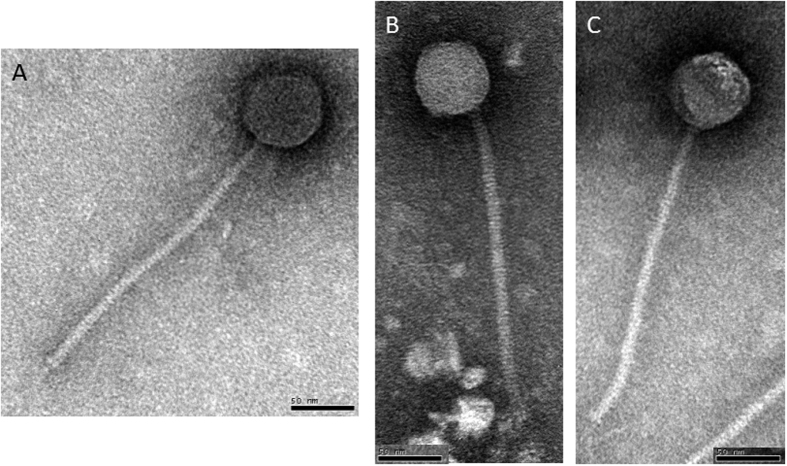
Electron micrographs of virulent *S. thermophilus* phages 53 (**A**), 73 (**B**), and 128 (**C**). Bars indicate 50 nm.

Uruguayan phages 53, 73, and 128 (for isolation details, see Methods) had a different and limited host range as they mostly infected only their Uruguayan host strains (Table 1). Of note, phage 73 could also infect the *S. thermophilus* reference strain SMQ-301, host of the model *cos*-type phage DT1^22^. The three Uruguayan phages were characterized by electron microscopy and, as expected, found to belong to the *Siphoviridae* family (Fig. 1, Table 2). Their capsid size and tail length were similar to those previously reported for other *S. thermophilus* phages (for review, see ref. 6). The three phages had different EcoRI and EcoRV restriction profiles (data not shown), confirming their uniqueness. Their structural protein profiles were similar to the reference *cos*-type phage DT1 (data not shown).

### Genome organization and comparison

**Figure 2:**
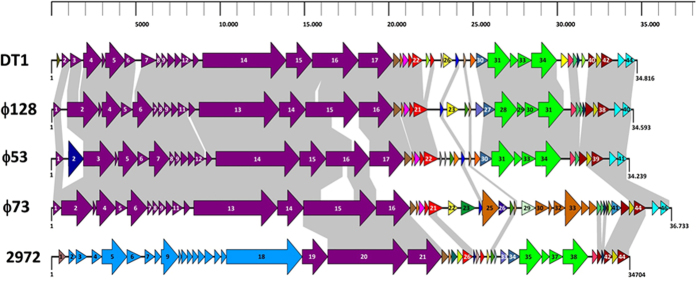
Graphical representation of *S. thermophilus* bacteriophage genomes. Each line represents a different phage genome and each arrow represents a putative protein. The graphical representation was made with XplasMap. The length of the genomes was converted from base pairs to pixels by measuring the output images from XplasMap in MS paint, then resized to maintain correspondence with the length of the genomes. The individual ORFs were aligned using MUSCLE after BLAST analysis. Conserved (>90% amino acid identity) deduced proteins are connected by grey shading, the only exception been between ORF21 of DT1 and ORF20 of 128 (79% identity). See Tables S1 to S3 for detailed genome annotations.

The genomes of the three Uruguayan phages were sequenced and annotated. The results of the annotation analysis are presented in Tables S1, S2 and S3 in Supplementary Materials. Phage 53 has a genome size of 34,239 bp (41 *orfs*), while phage 73 has a dsDNA genome of 36,733 bp (46 *orfs*) and that of phage 128 is 34,539 bp (40 *orfs*). The genome sizes of these three phages are in line with previously characterized *S. thermophilus* phages^6^. The GC content of the three genomes was 39%, which is similar to *S. thermophilus* host strains^22^. The three genomes are organized into modular regions as observed with other *S. thermophilus* phages^23^ and illustrated in Fig. 2.

Functions could be assigned for 57% (phage 73) to 83% (phage 128) of the deduced phage proteins (Tables S1–S3). No gene coding for an integrase was found in these three genomes, confirming their lytic lifestyle. Similar to other virulent *S. thermophilus* phages^24^, genes coding for putative cro-like transcriptional regulators were found in the three genomes. It may suggest that these phages are derived from prophages^23^. Cro-like regulators are also believed to play a role in the replication of these phages^25^. Also of interest was the presence of a gene coding for a putative adenine-specific methyltransferase (*orf23*) in the genome of phage 73, likely used to counteract restriction-modification systems (for review, see ref. 26). Overall, the deduced phages proteins are similar to those found in other *S. thermophilus* phages, although they were closer to each other (Tables S4, S5 and S6 in Supplementary Materials). Only ORF23 and ORF24 of phage 53 were unique and not found in public databases. Their functions are unknown.

### CRISPR analysis of the wild-type *S. thermophilus* phage sensitive strains

**Figure 3:**
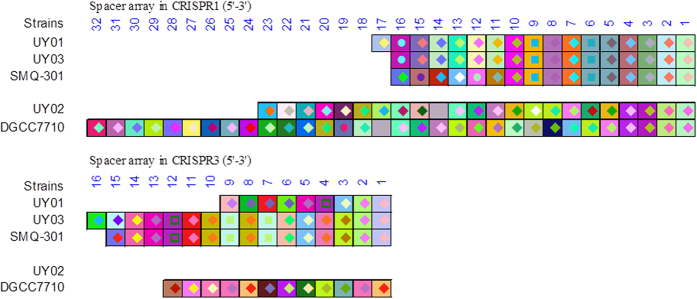
Graphic representation of the spacer organization in CRISPR1 and CRISPR3 of five *S. thermophilus* strains. The direct repeat sequences have been removed and only the spacer sequences are represented. The spacers are aligned and the direction of the spacers is shown 5′–3′, with respect to the leader sequence. Each spacer is represented by a combination of one select character in a particular font color, on a particular background color. The combination of character color and background color allows unique representation of a particular spacer.

Using a PCR approach and the sequencing of PCR products, the CRISPR arrays of the two active type II-A CRISPR-Cas systems (CR1 and CR3) were analyzed in the three *S. thermophilus* host strains (Fig. 3). The CR1 arrays of the wild-type strains Uy01, Uy02 and Uy03 were found to contain 17, 23, and 16 spacers, respectively (Tables S7, S8 and S9). Interestingly, the CR1 array of strain Uy01 shared 16 spacers with the CR1 of strain Uy03, indicating that they are related. The additional spacer in Uy01 was located at the array 5′-end, the hallmark feature for the acquisition of recent spacers in a CRISPR array^27^. BLAST of this unique 30-nt spacer showed a perfect match (30/30) to a section of *orf14* in the *S. thermophilus* prophage TP-J34 as well as *orf5* of *S. thermophilus* virulent phage 5093. Of note, the CR1 spacer content of Uy03 is identical to the CR1 of *S. thermophilus* strain LMD-9 and also shared 10 spacers with the CR1 of *S. thermophilus* SMQ-301. The virulent phage DT1, which infects LMD-9 and SMQ-301, can also infect strain Uy01, confirming their relatedness.

The CR1 array of strain Uy02 was more related to strain DGCC7710 as they shared 11 spacers (spacers 1–4, 7, 8, 11–14, 18 in Uy02 as compared to spacers 1–6, 14–18 in DGCC7710) (Table S9). Spacer acquisition, deletion or recombination appeared to have occurred within the CR1 arrays of these two related strains.

Interestingly, the CR1 spacer #12 of *S. thermophilus* Uy02 was almost identical (27 nucleotides out of 30) to a region (*orf6*) on the genome of its phage 128 (Table S8). Others have shown that a perfect nucleotide match (30/30) is needed to confer phage resistance^28^, hence the sensitivity of this strain to phage 128. A similar observation (28/30) was made with the spacer #1 of CR1 array of *S. thermophilus* Uy03 and the gene *orf16* of its phage 73 (Table S9).

The CR3 array was analyzed in these three *S. thermophilus* strains using the same approach. Attempts to amplify the CR3 locus in strain Uy02 were unsuccessful under the conditions used. On the other hand, the CR3 array of strains Uy01 and Uy03 were found to contain 9 and 16 spacers, respectively (Fig. 3, Tables S10 and S11). Again, the CR3 array of Uy01 was similar to the CR3 of strain LMD-9, with 8 identical spacers. The additional spacer in Uy01 was at the 5′-end of the CR3 array. The CR3 of Uy03 was closely related to the CR3 array of SMQ-301, as they shared 14 spacers. Differences were only found at the 5′-end of the array (Fig. 3).

Similar to CR1, spacers in CR3 regions were found to be highly similar to regions in the genome of Uruguayan phages. For example, the CR3 spacer #3 of Uy01 was similar (27/30 nucleotides) to a region in the *orf14* of phage 53 (Table S10) while spacers #5 and #16 had identities with regions of genes of *orf13* (27/30) and *orf15* (28/30), respectively.

### Selection and analysis of BIMs

One of the most efficient strategies to cope with virulent phages of *S. thermophilus* is to use phage-resistant strains^12^. This lactic acid bacterium appears to mostly rely on CRISPR-Cas systems to survive phage infection, specifically through the emergence of bacteriophage-insensitive mutants (BIMs)^27^. To verify if the CRISPR-Cas systems were active against Uruguayan phages, *S. thermophilus* strain Uy02 was challenged with the virulent phage 128. The strain Uy02 was selected because its CRISPR1 array is related to the CR1 of *S. thermophilus* DGCC7710, a well-known model strain used for spacer acquisition^16^,^20^,^21^,^27^,^28^,^29^,^30^,^31^,^32^,^33^,^34^,^35^,^36^,^37^.

**Figure 4:**
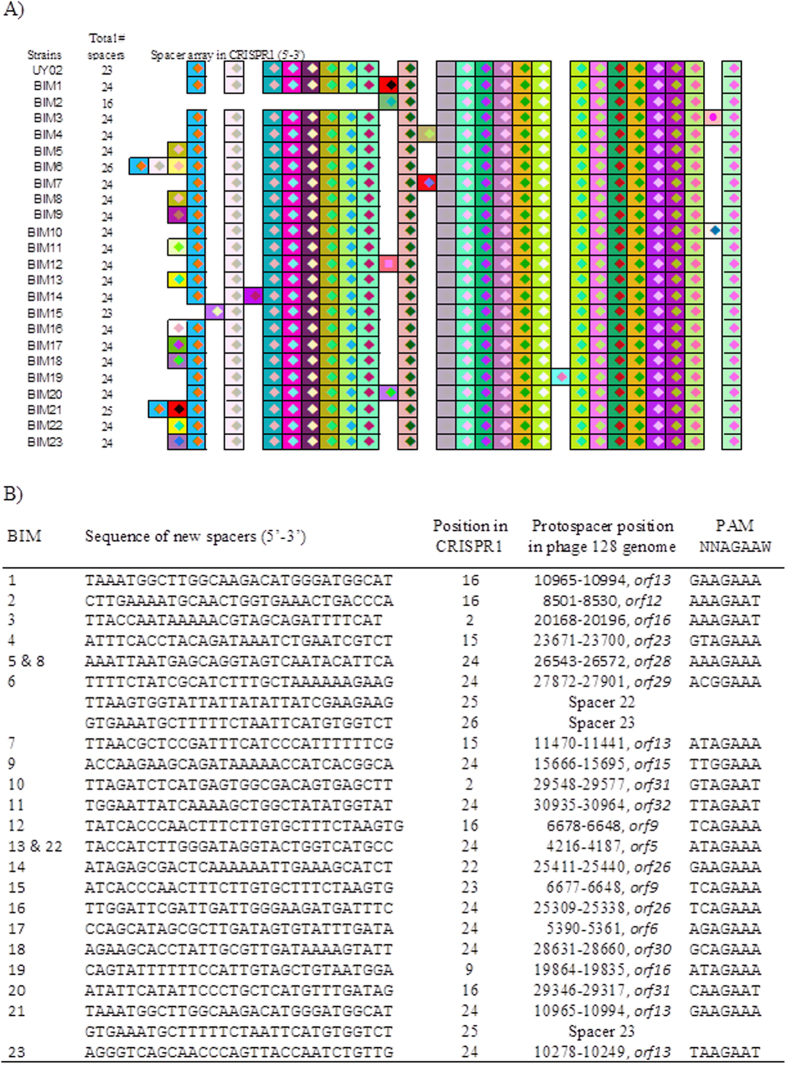
Graphic representation of the spacer organization in CRISPR1 of the wild-type phage-sensitive *S. thermophilus* UY02 (ancestral spacers) and 23 BIMs. (**A**) The direct repeat sequences have been removed and only the spacer sequences are represented. The spacers are aligned and the direction of the spacers is shown 5′–3′, with respect to the leader sequence. (**B**) Nucleotide sequence and position of newly acquired spacers in CRISPR1 of the BIMs obtained following a challenge with phage 128. The corresponding position of the protospacers in the phage genome, including the targeted gene and the PAM sequence, are also indicated.

Following the phage challenge assay, 23 BIMs were picked at random and their CRISPR1 array was sequenced and analyzed (Fig. 4). Surprisingly, only 12 BIMs out of the 23 had acquired a single new spacer at the 5′-end of the array. In two of these 12 BIMs, spacer deletion also occurred, deleting 1 (BIM15) or 8 (BIM2) spacers from the wild-type spacer array. Two other BIMs, out of the 23 analyzed, acquired two (BIM21) or three (BIM6) new spacers at the 5′-end, which included the duplication of spacer #23 (twice) and spacer #22 (once). More importantly, 9/23 BIMs have acquired a new spacer *within* the CRISPR array. Up until now, the latter was believed to be a rare event in *S. thermophilus*. These seemingly oddly added spacers were acquired between spacers 1 and 2 (BIM3 and BIM10), 8 and 9 (BIM19), 14 and 15 (BIM4 and BIM7), 15 and 16 (BIM1, BIM12, BIM20), as well as 21 and 22 (BIM14).

The sequence of all newly acquired spacers (except the duplicated ones) were found as protospacers in the genome of the phage used in the challenge assay, namely the virulent phage 128 (Fig. 4B). The protospacers in the phage were located in different positions of the genome but next to a consensus protospacer adjacent motif (PAM) sequence (NNAGAAW) (Fig. 4B). Of interest, all randomly picked BIMs have acquired different spacers.

### Analyses of the leader regions

**Figure 5:**
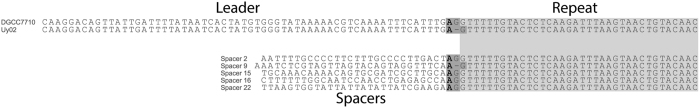
Nucleotide alignment of leader, spacer and repeat sequences upstream of newly acquired spacers. (Top) Nucleotide sequences of the leader and the first repeat of the CRISPR1 array in the wild-type strains *S. thermophilus* DGCC7710 and Uy02. (Bottom) Nucleotide sequences of the spacer and repeat upstream of the newly acquired ectopic spacer in the CRISPR1 of five BIMs of *S. thermophilus*.

Recently, two studies revealed the importance of the leader region upstream of the CRISPR array in the spacer acquisition process of type II systems of *S. thermophilus*^29^ and *Streptococcus pyogenes*^38^. In the latter case, it was shown that a conserved sequence directly upstream of the first repeat specified the site of new spacer integration and that engineered mutation of this sequence resulted in addition of new spacers into the middle of the CRISPR array^38^. Using a set of constructed strains in *S. thermophilus*, it was demonstrated that the nucleotide sequences close to the site of integration, in both the leader and repeat of the CRISPR array, are required for spacer acquisition^29^. Therefore, we compared the leader region between *S. thermophilus* strains Uy02 (spacer acquisition at the 5-end and within the CRISPR array) and DGCC7710 (spacer acquisition only at the 5′-end) (Fig. 5). We noticed the deletion of the last nucleotide (G) of the leader in the Uy02 strain as compared to DGCC7710.

We also investigated the spacer sequences upstream of all the repeats. Specifically, we investigated the spacer sequences upstream of the spacers that were acquired within the array in Uy02 (Fig. 4), mainly spacers 2, 9 15, 16 and 22 (Fig. 5). Interestingly, they all shared an adenine residue at the -2 position (from the 3′-end). The same adenine at position -2 was also found in the leader region of the array. A guanine residue was also present at the -1 position in most cases. It should be noted that in the two cases where the guanine residue was absent at the -1 position (leader and spacer #9), the first residue of the repeat was a guanine (Fig. 5).

## Discussion

The genome analysis of the three Uruguayan virulent phages characterized in this study showed that they share genetic elements found in phages isolated in Argentina as well as in Europe and North America. They do not seem to possess distinctive features as a group. Our analyses are consistent with previous studies, which indicated that *S. thermophilus* phages can be classified into only a few homogenous groups^1^. However, this seemingly lack of diversity may be the result of a biased towards the ecological niche. Most, if not all, *S. thermophilus* phages have been isolated from dairy environments^6^. Moreover, it is believed that relatively few truly distinct strains of *S. thermophilus* are used by the dairy industry worldwide, leading to the isolation of closely related virulent phages. The diversity of *S. thermophilus* strains used in the Uruguayan dairy industry is likely limited as local starter culture suppliers import many of them from larger multinational companies.

We analyzed the CRISPR1 and CRISPR3 of the three wild-type *S. thermophilus* hosts for the above three phages. The CRISPR1 array of strains Uy01 and Uy03 were highly similar to the CRISPR1 array of strains SMQ-301 and LMD-9, which are industrially used in Canada and the USA, respectively. The CRISPR3 array of strain Uy03 was also very similar to the CR3 of SMQ-301 while the CRISPR3 array of Uy01 was related to the CR3 array of LMD-9. On the other hand, the CRISPR1 array of strain Uy02 was related to *S. thermophilus* DGCC7710, heavily used in Europe.

One of the hallmark features of CRISPR-Cas systems is the integration of new spacer sequences into the 5′-end of the CRISPR locus following a phage challenge assay^38^,^39^. The polarity of spacer incorporation during the adaptation stage has been particularly well studied in the strain *S. thermophilus* DGCC7710. Interestingly, when we performed a phage challenge assay with the strain Uy02 and its virulent phage 128, we did not always observe new spacer addition in the first position of the CRISPR array. Out of the 23 distinct BIMs analyzed, 14 had acquired at least one spacer at the 5′-end while 9 BIMs had acquired a new spacer at five different positions within the Uy02 CRISPR array. The integration of new spacers into the middle of the CRISPR array was recently referred as ectopic spacer integration^38^.

Recent work^29^ demonstrated the key role of seven conserved nucleotide sequences (5′-ATTTGAG-3′) at the 3′-end of the leader sequence in the acquisition of new spacers in *S. thermophilus* CR1 array. Subsequently, it was shown that a deletion of the -5 to -1 leader region of *S. pyogenes* type II CRISPR system (cloned and tested in *Staphylococcus aureus*) resulted in ectopic spacer integration^38^. This region of the leader was coined the “leader-anchoring sequence” (LAS). In the case of strain *S. thermophilus* Uy02, the 3′-end of the leader sequence lacks a guanine at the position -1, which likely explains the atypical integration of spacers.

It was also shown recently that in the absence of an appropriate LAS, other sequences within the *S. pyogenes* type II CRISPR array can anchor spacer integration in *S. aureus*, particularly due to short sequences immediately upstream of the repeats^38^. We analyzed the sequences of the five spacers adjacent (5′-end) to the newly acquired spacer to see if a nucleotide motif was present to guide the acquisition of a new spacer within the array. We noticed that in all five adjacent spacers, an adenine was located at position -2 (3′-end) while a guanine was found at position -1 in 4 out of the 5 spacers. Interestingly, in the spacer with the missing guanine, the first nucleotide of the repeat is a guanine. Our data suggest that the LAS is perhaps limited to only a very few nucleotides, including the adenine at position -2.

While more studies are needed to understand the key role of the leader nucleotide sequence in the acquisition of new CRISPR spacers^40^, our results clearly indicate that wild-type strains of *S. thermophilus* are capable of natural ectopic spacer integration. It remains to be seen if other wild-type strains of *S. thermophilus* as well as of other microbial species are also capable of acquiring new spacers within the CRISPR array and provide phage resistance. It also suggests that studies on spacer acquisition using metagenomics or deep sequencing should not solely focus on the leader-proximal end of CRISPR cassette.

## Methods

### Phages and bacterial strains

Virulent phages 53, 73, and 128 were isolated from failed mozzarella productions in Uruguay. *S. thermophilus* host strains Uy01, Uy02, and Uy03 were obtained from a local starter culture supplier and were grown in LM17 medium at 42 °C. Reference phages DT1 (*cos*-type) and 2972 (*pac*-type), including their respective *S. thermophilus* hosts SMQ-301^10^ and DGCC7710^23^, were obtained from the Félix d’Hérelle Center (www.phage.ulaval.ca). Phages and bacterial strains were stored, as frozen stocks, in LM17 supplemented with 15% (v/v) glycerol. Phages were amplified in LM17 supplemented with 10 mM CaCl_2_ (LM17-CaCl_2_). Phage titers were determined as described elsewhere^16^. Each phage was plaque purified three times and concentrated through CsCl gradient^41^. Briefly, one liter of phage lysate wasemented with 10% polyethylene glycol (PEG 8000). PEG-concentrated phages were recovered by centrifugation and subjected to ultracentrifugation using a discontinuous CsCl gradient in a Beckman SW41 Ti rotor (35,000 rpm, 3 h at 20 °C). The phage band was retrieved and a second ultracentrifugation was performed with a continuous gradient of CsCl using a Beckman NVT65 rotor (60,000 rpm, 18 h at 20 °C). The purified phages were dialyzed in buffer (50 mM Tris-HCl pH 7.5, 100 mM NaCl, 8 mM MgSO_4_) and stored at 4 °C.

### Electron microscopy analysis

Phages were observed by electron microscopy as previously described^42^. Briefly, 1.5 mL of phage lysate was centrifuged at 23,500 g for 1 h at 4 °C and the pellet washed twice with NH_4_Ac (0.1 M, pH 7.0). The resulting phage preparation was then used to prepare observation grids, which were stained with phosphotungstic acid (2%, pH 7.0) and observed with a JEOL 1230 electron microscope at the Université Laval.

### Phage genome sequencing and analysis

Phage genomic DNA was isolated directly from phage lysate using the QIAGEN Lambda Mini kit. Sequencing librairies were prepared with the Nextera XT DNA Library Preparation Kit and then sequenced using paired-end (2 × 250 bp) on a MiSeq system with the MiSeq reagent kit v2 (Illumina). Assembly from reads was performed using Ray Assembler. To complete and confirm the genomes, sequencing was carried out using an ABI Prism 3100 Genetic Analyzer at the Plateforme de séquençage et de génotypage des génomes du Centre Hospitalier de l’Université Laval. Contig assembly and editing were carried out using PreGap and Gap4 from Staden Package^43^. Sequence analysis was performed using the software ORF Finder for open reading frames prediction (http://www.ncbi.nlm.nih.gov/gorf/gorf.html), BLASTp for protein sequence comparison (http://blast.ncbi.nlm.nih.gov/Blast.cgi), and the ApE suite for DNA editing and annotations (ApE v2.0.45, http://biologylabs.utah.edu/jorgensen/wayned/ape/). An ORF was considered only if its starting codon was AUG, UUG or GUG and possessed at least 30 amino acids (aa). The search for a ribosomal binding site (RBS) similar to standard Shine-Dalgarno sequence was also carried out. Theoretical molecular masses (MM) and isoeletric points (pI) of the phage proteins were obtained using ProtParam (http://web.expasy.org/protparam/).

### *S. thermophilus* CRISPR loci analysis

CRISPR loci CR1 and CR3 were amplified by PCR (Feldan Taq DNA Polymerase) and then sequenced. CRISPR1 analysis was performed with the primers yc70 (5-TGCTGAGACAACCTAGTCTCTC-3) and CR1-rev (5-TAAACAGAGCCTCCCTATCC-3) whereas primers CR3-fwd (5-CTGAGATTAATAGTGCGATTACG-3) and CR3-rev (5-GCTGGATATTCGTATAACATGTC-3) were used for the amplification of CRISPR3^44^. Spacer analyses were performed with Geneious version (7.1.8)^45^.

### Bacteriophage-Insensitive Mutants (BIMs)

BIMs were obtained by infecting the sensitive strain UY02 with phage 128. Briefly, approximately 5 × 10^8^ CFU of *S. thermophilus* cells were mixed with 1 × 10^8^ PFU of phages in 4 ml of soft LM17-CaCl_2_ (0.75% agar) and poured on a LM17-CaCl_2_ agar plate (1.5% agar). Plates were incubated 48 hours at 42 °C. Individual colonies were recovered, streaked (three times), and colonies incubated overnight in LM17 broth. The cultures were then tested for phage resistance as described elsewhere^28^.

## Additional Information

**Accession codes:** The annotated genomic sequence of phages 53, 73 and 128 were deposited in GenBank under accession numbers KT717084, KT717083, and KT717085 respectively. Raw reads have been submitted to the sequence read archive under the accession numbers SRR5124160 (phage 53), SRR5124161 (phage 73), and SRR5124159 (phage 128).

**How to cite this article:** Achigar, R. *et al*. Phage-host interactions in *Streptococcus thermophilus*: Genome analysis of phages isolated in Uruguay and ectopic spacer acquisition in CRISPR array. *Sci. Rep.* **7**, 43438; doi: 10.1038/srep43438 (2017).

**Publisher's note:** Springer Nature remains neutral with regard to jurisdictional claims in published maps and institutional affiliations.

## Supplementary Material

Supplementary Tables

## Figures and Tables

**Table 1:** Host range of five phages of *S. thermophilus*.

Phages	*S. thermophilus* strains				
	Uy01	Uy02	Uy03	SMQ-301	DGCC7710
53	+	−	−	−	−
73	−	−	+	+	−
128	−	+	−	−	−
DT1	+	−	+	+	−
2972	−	−	−	−	+

+Positive infection.

−Negative infection.

**Table 2:** Phage dimensions of three new uruguayan phages.

Phage	Dimensions (nm)		
	Capsid	Tail length	Tail width
53	65.9+/−2.7	249.4+/−6.1	11.0+/−0.6
73	65.5+/−1.9	244.9+/−9.3	11.2+/−0.5
128	67.2+/−2.9	251.7+/−4.7	10.9+/−0.6
