# Supplementary material for: Phage-host interactions in Streptococcus thermophilus: Genome analysis of phages isolated in Uruguay and ectopic spacer acquisition in CRISPR array
Source: Sci Rep. 2017 Mar 6;7:43438. doi: 10.1038/srep43438 (PMC5338259; doi:10.1038/srep43438)
Supplement: Supplementary Tables [file srep43438-s1.pdf]

SUPPLEMENTARY TABLES

Phage-host interactions in *Streptococcus thermophilus*: Genome analysis of phages isolated in Uruguay and ectopic spacer acquisition in CRISPR array

Rodrigo Achigar[a], Alfonso H. Magadán[b†], Denise M. Tremblay[b],

María Julia Pianzzola[a], and Sylvain Moineau[b*]

[a] Laboratorio de Microbiología Molecular, Departamento de Biociencias, Facultad de Química, Universidad de la República, Montevideo, Uruguay

[b] Département de Biochimie, de Microbiologie et de Bioinformatique & PROTEO, Faculté des Sciences et de Génie, Félix d'Hérelle Reference Center for Bacterial Viruses & GREB, Faculté de Médecine Dentaire, Université Laval, Québec City, Québec, Canada, G1V 0A6.

* Corresponding author. Mailing address: Département de Biochimie, de Microbiologie et de Bioinformatique, Faculté des Sciences et de Génie Université Laval, Québec, Canada, G1V 0A6.

Phone: 1-418-656-3712. E-mail: Sylvain.Moineau@bcm.ulaval.ca

Present address[†]: Department for Strains, Chr. Hansen A/S, 2970 Hørsholm, Denmark.

TABLE S1: Phage 53 ORFs and general features

ORF	Start	Stop	%GC	Size (aa)	MW (kDa)	pI	RBS **AAAGGAGGTGA**	Protein function or similarity	BLAST match	% identity (aa)	Accession No.
1	296	757	40	153	17.42	6.8	**AACGGAGAGGA**GTAATGATGA***GTG***	Terminase small subunit	ORF2, DT1	144/151(95%)	NP_049390.1
2	1030	1938	35	302	35.9	9	**AAAGGGCAAAA*ATG***	-	Hyp protein, *B. cereus*	125/305(41%)	WP_002192091.1
3	1919	3793	40	624	71.9	4.6	**AAAGGAGGTGC*TTG***	Terminase large subunit	ORF22, 7201	526/623(84%)	NP_038323.1
4	3797	3976	37	59	6.6	9.8	**AGAGGAGTATT**AATAT***ATG***	Head-tail joining protein	ORF5, DT1	59/59(100%)	NP_049393.1
5	3994	5154	41	386	42.7	5	**AAAGGAGGTGA**TAACAA***TTG***	Portal protein	ORF6, DT1	383/386(99%)	NP_049394.1
6	5141	5809	38	222	24.5	4.7	**AAAGGAGGTGA**GATAA***ATG***	Scaffolding protein	ORF5, Abc2	222/222(100%)	YP_003347414.1
7	5824	7017	38	397	44.1	4.9	**AAAGGAAAATA**ATTA***ATG***	Major capsid protein	ORF6, Abc2	393/397(99%)	YP_003347415.1
8	7032	7346	44	104	11.6	4	**TTAGGAGGTAA**GCT***ATG***	DNA packaging protein	ORF9, DT1	104/104(100%)	NP_049397.1
9	7346	7696	40	116	13.3	9.9	**GAAAGAGGTGA**CTA***ATG***	Head-tail joining protein	ORF10, DT1	116/116(100%)	NP_049398.1
10	7703	8125	43	140	15.6	10	**AAGTTGGGTGA**TAGCTT***ATG***	Tail protein	ORF11, DT1	137/140(98%)	NP_049399.1
11	8130	8501	32	123	14.1	4.2	**AAGGGAGGGGA**GTAATTAA***GTG***	Tail protein	ORF12, DT1	119/123(97%)	NP_049400.1
12	8520	9128	40	202	21.9	6.5	**AAAGGAGAAAA**TATAT***ATG***	Major tail protein	ORF11, Abc2	197/203(97%)	YP_003347420.1
13	9202	9555	37	117	13.5	4.3	**AAAGGAGTAAA**GACCACA***ATG***	Tail protein	ORF14, DT1	114/117(97%)	NP_049402.1
14	9774	14744	42	1656	182.8	10	**AAAGGAGGGAA**TATAAC***ATG***	Tail protein	ORF15, DT1	1600/1656(97%)	NP_049403.2
15	14741	16300	40	519	58.5	5.9	**TTAGGAGGTCA**AATTAT***TTG***	Tail protein	ORF15, Abc2	517/519(99%)	YP_003347424.1
16	16300	18885	39	861	97.8	4.8	**GAAGGAGCGCT**TTGTTTA***ATG***	Host specificity protein	ORF18, Abc2	780/861(91%)	AAK83243.1
17	18886	20934	42	682	75	6.2	**GTAGGAGGTTT**TTAA***TTG***	Tail protein	ORF21, 2972	538/660(82%)	YP_238504
18	20960	21370	34	136	15.7	4.5	**AAAGGAATAAT**T***ATG***	-	ORF20, ALQ13.2	135/136(99%)	YP_003344866.1
19	21390	21536	30	48	5.6	9.8	**AAAGGATAAAA**AGAT***ATG***	-	ORF22, DT1	48/48(100%)	NP_049410.1
20	21554	21877	36	107	12.3	6.5	**ATAGGAGGGAT**GTGTT***ATG***	-	ORF22, ALQ13.2	93/103(90%)	YP_003344868.1
21	21874	22116	37	80	8.9	9.8	**AGAGGATAATA**ATAAA***ATG***	-	ORF24, DT1	78/80(98%)	NP_049412.1
22	22118	22963	41	281	31.3	4.2	**AAAGGAGAAAT**AAA***ATG***	Lysin	ORF22, Abc2	257/281(91%)	YP_003347431.1
23	23057	23179	35	40	4.7	7.4	**GAAGCCTCAGC**ATT***ATG***	-	-	-	-
24	23348	23445	32	31	3.7	11.7	**ATAAGTGGTAA**TATAA***TTG***	-	-	-	-
25	23650	23877	38	75	8.6	9.9	**AAAGGAGATAA**CCT***ATG***	Cro repressor	ORF44, 5093	71/75(95%)	YP_002925127.1
26	23895	24185	36	96	11.2	7.3	**AAAGGAACAAT*ATG***	-	Hyp protein, *S. agalactiae*	64/90(71%)	WP_001156317.1
27	24328	24531	35	67	7.6	9.7	**AGAGGAGGAAC**AAAA***ATG***	Cro-like regulator	ORF5, TP-J34	59/67(88%)	YP_007392252.1
28	24783	24917	36	44	5.2	10.8	**AAAGGAATTTA**AAAA***ATG***	-	ORF32, ALQ13.2	43/46(93%)	YP_003344878.1
29	25119	25445	34	108	12.8	4.6	**AAAGTATCAAC**T***ATG***	-	ORF31, DT1	104/104(100%)	NP_049419.1
30	25449	26150	43	233	26.4	4.7	**AAAGGAAGAAA**TAACGG***ATG***	-	ORF32, DT1	233/233(100%)	NP_049420.1
31	26125	27456	40	443	50.4	9	**AAATTTGGTGA**TTTAG***ATG***	Helicase	ORF33, DT1	439/443(99%)	NP_049421.1
32	27463	27918	36	151	17.3	4.9	**TATGGAGATAA**AAAACT***ATG***	-	ORF34, DT1	150/151(99%)	NP_049422.1
33	27921	28736	40	271	30.5	7.9	**ACCTTCCGTTC**TAATT***ATG***	Replication protein	ORF35, DT1	271/271(100%)	NP_049423.1
34	28717	30240	36	507	59.2	7.6	**AAATAAGGAGG**A***TTG***	Primase	ORF38, 2972	492/505(97%)	YP_238521.1
35	30777	31100	38	107	12.2	10.3	**AAAGGAGATGT*ATG***	-	ORF38, DT1	105/107(98%)	NP_049426.1
36	31081	31329	37	82	9.7	9	**CTATGAGGATA**GTTG***ATG***	-	ORF39, DT1	82/82(100%)	NP_049427.1
37	31314	31790	39	158	18.5	4.7	**GAAAGAGATGG**TAGAACT***ATG***	DNA binding protein	ORF42, 2972	103/109(94%)	YP_238525.1
38	31759	32067	41	102	11.2	9.8	**AAAGGAAAGAT**GGTAA***ATG***	-	ORF24, TP-J34	76/104(73%)	YP_007392271.1
39	32064	32771	37	235	27.7	9.7	**AAAGGAAGAGG**GCA***ATG***	-	ORF45, Abc2	235/235(100%)	YP_003347454.1
40	33105	33524	38	139	16.5	9.8	**AAATTATTATA**CC***ATG***	-	ORF45, DT1	132/132(100%)	NP_049433.1
41	33597	34145	46	182	21.2	10.2	**TCAGAAGGACA**CAGTA***GTG***	HNH endonuclease	ORF46, DT1	180/185(97%)	NP_049434.1

*1: 1-689: 557/751(74%); 445-860: 210/464(45%)

TABLE S2: Phage 73 ORFs and general features

ORF	Start	Stop	%GC	Size (aa)	MW (kDa)	pI	RBS **AAAGGAGGTGA**	Protein function or similarity	BLAST match with *S. thermophilus* phages	% identity (aa)	Accession No.
1	87	459	39	152	17	5.1	**AACGGAGAGGA**GTAATGATGA***GTG***	Terminase small subunit	ORF152, Sfi21	145/152(95%)	NP_049966.1
2	567	2438	41	623	71.5	4.7	**AAAGGAGCAAC**A***GTG***	Terminase large subunit	ORF2, Abc2	611/623(98%)	YP_003347411.1
3	2442	2621	37	59	6.6	8.1	**ATTAGAGGAGT**ATTAATAT***ATG***	Head-tail joining protein	ORF3, Abc2	59/59(100%)	YP_003347412.1
4	2639	3799	41	368	42.7	5.1	**AAAGGAGGTGA**TAACAA***ATG***	Portal protein	ORF4, Abc2	384/386(99%)	YP_003347413.1
5	3786	4454	38	222	24.5	4.9	**AAAGGAGGTGA**GATAA***ATG***	Scaffolding protein	ORF7, DT1	222/222(100%)	NP_049395.1
6	4469	5662	38	397	44.2	5.1	**AAAGGAAAATA**ATTA***ATG***	Major capsid protein	ORF6, Abc2	393/397(99%)	YP_003347415.1
7	5677	5991	44	104	11.6	4.2	**TTAGGAGGTAA**GCT***ATG***	DNA packaging protein	ORF9, DT1	104/104(100%)	NP_049397.1
8	5991	6341	40	116	13.5	9.6	**GAAAGAGGTGA**CTA***ATG***	Head-tail joining protein	ORF8, Abc2	115/116(99%)	YP_003347417.1
9	6348	6770	43	140	15.6	9.4	**AAGTTGGGTGA**TAGCTT***ATG***	Tail component protein	ORF11, DT1	137/140(98%)	NP_049399.1
10	6775	7146	32	123	14	4.4	**AAGGGAGGGGA**GTGATTAA***GTG***	Tail component protein	ORF12, DT1	119/123(97%)	NP_049400.1
11	7165	7773	40	202	21.8	6.1	**AAAGGAGAAAA**TATAT***ATG***	Major tail protein	ORF11, Abc2	197/203(97%)	YP_003347420.1
12	7847	8200	37	117	13.5	4.5	**AAAGGAGTAAA**GACCACA***ATG***	Tail component protein	ORF14, DT1	114/117(97%)	NP_049402.1
13	8419	13389	41	1656	183.2	9.3	**AAAGGAGGGAA**TATAAC***ATG***	Tail component protein	ORF15, DT1	1591/1656(96%)	NP_049403.2
14	13386	14945	40	519	58.4	5.6	**TTAGGAGGTCA**AATTAT***TTG***	Tail component protein	ORF15, Abc2	514/519(99%)	YP_003347424.1
15	14924	19234	41	1436	159.6	5.1	**CCAACAATTGA**AATTTC***ATG***	Receptor binding protein	ORF18, MD2	85%	AAK83242.1
16	19235	21241	42	668	73	6.3	**GTAGGAGGTTT**TTAA***TTG***	Minor tail protein	2972	592/673(88%)	YP_238504.1
17	21257	21604	33	115	13.3	4.7	**AAAGAAGGAAA**ATTC***ATG***	-	ORF50, TP-J34	110/115(96%)	YP_007392297.1
18	21624	21770	34	48	5.4	9.5	**GAAAGAGGAAA**AAGAT***ATG***	-	ORF51, TP-J34	46/48(96%)	YP_007392298.1
19	21788	22111	35	107	12.5	5.8	**ATAGGAGGGAT**GTGTT***ATG***	-	ORF22, ALQ13.2	106/107(99%)	YP_003344868.1
20	22119	22361	36	80	8.9	8.1	**TGAGAGGATGA**AGAATAA***ATG***	Holin	ORF23, ALQ13.2	79/80(99%)	YP_003344869.1
21	22363	23208	42	281	31.2	4.3	**AAAGGAGAAAT**AAA***ATG***	Lysin	ORF22, Abc2	263/281(94%)	YP_003347431.1
22	23526	24077	38	183	21.4	7.7	**GGGAGAGGTAA**ACAAA***ATG***	-	ORF27, ALQ13.2	171/183(93%)	YP_003344873.1
23	24284	25156	32	290	33.5	5.2	**AGAGAGGGATT**TA***ATG***	Adenine specific methyltransferase	*	285/290(98%)	CAB46541.1
24	25333	25536	38	67	7.7	9.7	**GAAGGAGGAAC**AAA***ATG***	Cro-like regulatory protein	ORF23, Abc2	60/67(90%)	YP_003347432.1
25	25526	26521	37	331	38.7	5.5	**AAATCGTCTGA**TTTGT***ATG***	-	ORF45, 5093	194/284(68%)	YP_002925128.1
26	26514	27053	35	179	20.7	8.8	**GAAGGAGAAAT**CATCA***ATG***	-	ORF46, 5093	162/166(98%)	YP_002925129.1
27	27180	27389	28	69	8.2	8.8	**AAAGGAGAAAC**GA***ATG***	Cro-like repressor	ORF31, ALQ13.2	68/69(99%)	YP_003344877.1
28	27422	27565	31	47	5.5	9.8	**TAGAGAGGAAT**CAAAA***ATG***	-	ORF29, Abc2	43/47(91%)	YP_003347438.1
29	27877	28686	33	269	31.5	7.7	**AAAGAGAGGGA**TAAGATTA***ATG***	-	ORF4, 7201	235/269(87%)	NP_038304.1
30	28699	29481	39	260	30.5	8.2	**CTAAGAGGTTC**TTTAT***ATG***	DnaC like protein	ORF5, 7201	233/260(90%)	NP_038305.1
31	29478	29660	36	60	7.3	6.2	**CAAGAGGATGA**TGTT***ATG***	-	ORF32, Abc2	60/60(100%)	YP_003347441.1
32	29788	30444	37	218	25	6.2	**AAAAGAGGATA**TGAC***ATG***	ERF like protein	ORF33, Abc2	217/218(99%)	YP_003347442.1
33	30447	31418	39	323	37.7	5.1	**AACGGAAGGGA**TAAAT***ATG***	-	ORF34, Abc2	323/323(100%)	YP_003347443.1
34	31415	31867	42	150	17	5.8	**AAAGGAGAAAA**CAA***ATG***	Single-stranded DNA binding protein	ORF35, Abc2	149/150(99%)	YP_003347444.1
35	31877	32338	38	153	18	9.5	**TAAGGTGAAAC**T***ATG***	-	ORF36, Abc2	153/153(100%)	YP_003347445.1
36	32336	32571	37	78	9.2	6.1	**CAAGGAGTTGG**A***ATG***	-	ORF37, Abc2	72/78(92%)	YP_003347446.1
37	32562	32735	41	57	6.5	7.9	**GAAAGAGATGA**TAGAACT***ATG***	-	ORF42, 858	53/57(93%)	YP_001686836.1
38	32732	32887	28	51	6.3	6.0	**GTAGGAGATTA**GTAGAGTT***ATG***	-	ORF40, DT1	48/51(94%)	NP_049428.1
39	32898	33011	37	37	4	5.8	**ATAGATGGCAA**GAT***ATG***	-	ORF20, TP-J34	20/25(80%)	YP_007392267.1
40	33016	33228	34	70	8	5.2	**GAAGGGATAGA**ATA***ATG***	-	ORF41, DT1	69/70(99%)	NP_049429.1
41	33215	33763	36	182	33.7	5.2	**CTAGGAGAAGA**AA***ATG***	-	ORF22, TP-J34	131/181(72%)	YP_007392269.1
42	33764	34270	38	168	19.1	5.4	**GACAGAGGTGG**AATAG***ATG***	DNA binding protein	ORF43, Abc2	158/168(94%)	YP_003347452.1
43	34245	34547	39	100	11.1	9.4	**AAAGGAATAAT**GATTG***ATG***	-	ORF43, DT1	99/100(99%)	NP_049431.1
44	34544	35251	38	235	27.5	9.2	**AAAGGAAGAGG**GCA***ATG***	-	ORF236, Sfi11	195/233(84%)	NP_056722.1
45	35611	36009	39	132	15.5	8.9	**AAAGGAAAGAC**AATTT***ATG***	-	ORF45, DT1	132/132(100%)	NP_049433.1
46	36121	36639	47	172	20	9.7	**AGAGGAGGGAA**GCCA***ATG***	HNH endonuclease	ORF48, Abc2	172/172(100%)	YP_003347457.1

*BLAST result match *Streptococcus thermophilus* genome

TABLE S3: Phage 128 ORFs and general features

ORF	Start	Stop	%GC	Size (aa)	MW (kDa)	pI	RBS **AAAGGAGGTGA**	Protein function or similarity	BLAST match with streptococcal phages*	% identity (aa)	Accession No.
1	88	549	42	153	17.3	8.7	**AGAGGAGAAAC**GATGA***GTG***	Terminase small subunit	ORF2, DT1	138/153 (90%)	NP_049390.1
2	902	2773	41	623	71.5	4.8	**AAAGGGGGTGA**TTAATAGTAA***ATG***	Terminase large subunit	ORF2, Abc2	607/623 (97%)	YP_003347411.1
3	2777	2956	37	59	6.6	8.2	**AGAGGAGTATT**AATAT***ATG***	Head-tail joining	ORF3, Abc2	59/59 (100%)	YP_003347412.1
4	2974	4134	41	386	42.7	5.1	**AAAGGAGGTGA**TAACAA***ATG***	Portal	ORF4, Abc2	384/386 (99%)	YP_003347413.1
5	4121	4789	38	222	24.5	5.0	**AAAGGAGGTGA**GATAA***ATG***	Scaffolding, Clp protease-like	ORF5, Abc2	222/222 (100%)	YP_003347414.1
6	4804	5997	39	397	44.1	5.1	**AAAGGAAAATA**ATTA***ATG***	Major capsid protein	ORF6, Abc2	389/397 (98%)	YP_003347415.1
7	6012	6326	44	104	11.5	4.3	**TTAGGAGGTAA**GCT***ATG***	Packaging	ORF9, DT1	104/104 (100%)	NP_049397.1
8	6326	6676	40	116	13.4	9.7	**GAAAGAGGTGA**CTA***ATG***	Capsid-tail joining	ORF8, Abc2	115/116 (99%)	YP_003347417.1
9	6683	7105	43	140	15.6	9.5	**AAGTTGGGTGA**TAGCTT***ATG***	Tail protein, DUF 646 superfamily	ORF11, DT1	137/140 (98%)	NP_049399.1
10	7110	7482	32	123	14.0	4.5	**GAGGGGAGTGA**TTAA***GTG***	Tail protein, DUF806	ORF12, DT1	117/123 (95%)	NP_049400.1
11	7500	8111	39	203	22.0	5.7	**AAAGGAGAAAA**TATAT***ATG***	Major tail protein	ORF13, DT1	196/203 (97%)	NP_049401.1
12	8144	8539	37	131	15.2	4.9	**CAAAGAGGTCA**GGCTT***ATG***	Tail protein, DUF 1268 superfamily	ORF14, DT1	111/117 (95%)	NP_049402.1
13	8758	13533	42	1591	175.0	9.2	**AAAGGAGGGAA**TATAAC***ATG***	Minor tail protein	ORF1560 gp, Sfi21	1293/1591 (81%)	NP_049978.1
14	13530	15095	38	521	58.7	5.5	**TTAGGAGGTCA**AATTAT***TTG***	Minor tail protein	YMC-2011	433/518 (84%)	YP_006561276.1
15	15091	18274	39	1059	119.1	5.3	**GAAGGAGCGTT**TTGTATA***ATG***	Receptor binding protein	ORF18, MD2	564/796 (71%)	AAK83242.1
16	18274	20322	41	682	76.3	6.1	**GTAGGAGGTGC**ATA***ATG***	Structural protein	ORF19, DT1	539/684 (79%)	NP_049407.2
17	20343	20771	35	142	16.4	4.7	**AAATGAGGAAT**GAAAAAAT***ATG***	DUF 1366 superfamily	ORF21, DT1	108/132 (82%)	NP_049409.1
18	20797	20943	35	48	5.5	9.4	**AAAGGGAAAAA**GAT***ATG***	-	ORF41, 7201	40/48 (83%)	NP_038342.1
19	20961	21284	35	107	12.5	6.1	**ATAGGAGGGAT**GTGTT***ATG***	-	ORF20, Abc2	103/107 (96%)	YP_003347429.1
20	21292	21534	38	80	9.8	5.5	**TTGAGAGGATA**ATAATAAA***ATG***	Holin	ORF21, Abc2	70/80 (88%)	YP_003347430.1
21	21536	22381	41	281	31.2	4.3	**AAAGGAAGGAA**AATAGT***ATG***	Endolysin	ORF44, 7201	252/281 (90%)	NP_038345.1
22	23103	23306	36	67	7.6	9.3	**GAAGGAGGAAC**AAA***ATG***	Cro-like regulatory protein	ORF23, Abc2	67/67 (100%)	YP_003347432.1
23	23518	24210	35	230	25.1	9.3	**CTGGGAGGAGA**ACAAAAA***ATG***	Putative protease	phiNJ2	44/118 (37%)	YP_006990374.1
24	24577	24786	28	69	8.2	8.9	**AAAGGAGAAAC**GA***ATG***	Cro represor	ORF31, ALQ13.2	69/69 (100%)	YP_003344877.1
25	24820	24954	35	44	5.2	10.0	**TAGAGAGGAAC**CAAAA***ATG***	-	ORF3, 7201	39/47 (83%)	NP_038303.1
26	25228	25701	36	157	18.4	6.1	**AGGGTAGGAAT**TAAAT***ATG***	gp157 superfamily	YMC-2011	124/157 (79%)	YP_006561241.1
27	25698	26372	45	224	25.4	5.0	**AAAGGAGAAAC**CTTAACATAAG***ATG***	NTP-binding motif protein	ORF32, DT1	214/222 (96%)	NP_049420.1
28	26362	27693	40	443	50.2	7.7	**AGAAGAGGTCT**TCAATT***TTG***	Helicase	ORF33, DT1	415/443 (94%)	NP_049421.1
29	27700	28155	36	151	17.3	4.9	**TATGGAGATAA**AAAACT***ATG***	DUF 669 superfamily	ORF36, ALQ13.2	151/151 (100%)	YP_003344882.1
30	28158	28973	41	271	30.5	5.4	**AATTGACCTTC**CATTCTAATT***ATG***	Replication protein	ORF271, Sfi21	267/271 (99%)	NP_050001.1
31	28960	30471	37	503	58.8	7.7	**TAAGGAGGATT**GGAC***TTG***	Primase	ORF36, DT1	487/491 (99%)	NP_049424.1
32	30898	31221	38	107	12.1	9.6	**TTGCGAGCATT**TATAAGGA***ATG***	VRR_NUC domain	ORF38, DT1	99/107 (93%)	NP_049426.1
33	31202	31444	37	80	9.5	9.1	**ACTGGAGATAG**TTG***ATG***	-	ORF39, DT1	71/79 (90%)	NP_049427.1
34	31435	31608	36	57	6.5	9.5	**GAAAGAGATGA**TAGAACT***ATG***	-	ORF38, Abc2	49/57 (86%)	YP_003347447.1
35	31605	31760	26	51	6.3	6.7	**GAAGGAGATTA**GTAGATTT***ATG***	-	ORF43, 2972	47/51 (92%)	YP_238524.1
36	31761	32258	37	165	19.0	5.5	**GGTTGAGGTAG**AATAA***ATG***	DNA binding protein	ORF42, DT1	160/165 (97%)	NP_049430.1
37	32230	32511	39	93	10.4	9.6	**AAAGGAGTAAT**GA***TTG***	DUF 1372	ORF42, ALQ13.2	71/98 (72%)	YP_003344888.1
38	32508	33215	40	235	28.0	9.4	**AAAGGGAGGGG**ACA***ATG***	-	ORF43, ALQ13.2	226/235 (96%)	YP_003344889.1
39	33482	33880	37	132	15.4	9.3	**AAAGGAAAGAC**AATTT***ATG***	DUF 1492 superfamily	ORF132, Sfi19	131/132 (99%)	NP_049923.1
40	33990	34499	48	169	19.5	9.5	**AAAGGAGGCAT**GCCA***ATG***	HNH endonuclease	ORF20, 7201	161/172 (94%)	NP_038321.1

* All *S. thermophilus* phages as well as *S. salivarius* YMC-2011 and *S. suis* phiNJ2

*2: 1-501:471/501(94%); 679-1059: 301/381(79%)

TABLE S4: Phage 53 proteins comparition against other uruguayan phages*

ORF	BLAST aganst Uruguayan phages	% Identity (aa)
1	ORF1,128	143/151(95%)
2	-	-
3	ORF2, 73	512/624(82%)
4	ORF3, 73 & 128	57/59(97%)
5	ORF4, 107	378/386(98%)
6	ORF5, 128	222/222(100%)
7	ORF6, 73	396/397(99%)
8	ORF7, 73 & 107 & 128	104/104(100%)
9	ORF8, 73	114/116(98%)
10	ORF9, 73 & 107	140/140(100%)
11	ORF10, 73	123/123(100%)
12	ORF11, 73	202/202(100%)
13	ORF12, 73	117/117(100%)
14	ORF13, 73	1631/1656(98%)
15	ORF14, 73	514/519(99%)
16	ORF15, 107	*2
17	ORF16, 73	539/565(95%)
18	ORF21, 93	115/135(85%)
19	ORF22, 93	47/48(98%)
20	ORF19, 73	92/103(89%)
21	ORF20, 73	77/80(96%)
22	ORF21, 73	254/281(90%)
23	-	-
24	ORF25, 107	28/31(90%)
25	ORF26, 107	75/75(100%)
26	ORF34, 93	90/94(96%)
27	ORF33, 93	61/67(91%)
28	ORF25, 128	40/44(91%)
29	-	-
30	ORF27, 128	214/222(96%)
31	ORF28, 128	415/443(94%)
32	ORF29, 128	147/151(97%)
33	ORF30, 128	264/271(97%)
34	ORF31, 128	481/502(96%)
35	ORF32, 128	101/107(94%)
36	ORF33, 128	71/79(90%)
37	ORF43, 107	98/109(90%)
38	ORF44, 107	87/98(89%)
39	ORF38, 128	198/235(84%)
40	ORF45,	132/132(100%)
41	ORF46, 73	168/172(98%)

*: in gray are proteins with higher similarity with proteins of other uruguayan phages

*2: 1-689: 557/751(74%); 445-860: 210/464(45%)

TABLE S5: Phage 73 proteins comparition against other uruguayan phages*

ORF	BLAST aganst Uruguayan phages	% Identity (aa)
1	ORF1 107	138/152(90%)
2	ORF3, 53	512/624(82%)
3	ORF3, 128	59/59(100%)
4	ORF5, 53	380/386(98%)
5	ORF5 107	222/222(100%)
6	ORF7, 53	396/397(99%)
7	ORF8, 53	104/104(100%)
8	ORF8, 128	116/116(100%)
9	ORF10, 53	140/140(100%)
10	ORF11, 53	123/123(100%)
11	ORF12, 53	202/202(100%)
12	ORF13, 53	117/117(100%)
13	ORF14, 53	1643/1656(99%)
14	ORF15, 53	515/519(99%)
15	ORF16, 53	471/543(86%)
16	ORF17, 53	547/565(96%)
17	ORF18, 53	108/136(79%)
18	ORF22, 93	41/48(85%)
19	ORF19,	105/107(98%)
20	ORF21, 53	78/80(97%)
21	ORF22, 53	267/281(95%)
22	ORF30, 93	181/183(98%)
23	-	-
24	ORF33, 93	66/67(98%)
25	ORF34, 93	198/219(90%)
26	ORF6, 128	42/100(42%)
27	ORF24, 128	68/69(98%)
28	ORF28, 53	40/47(85%)
29	ORF31, 107	263/269(97%)
30	ORF39, 93	248/260(95%)
31	ORF40, 93	60/60(100%)
32	ORF41, 93	217/218(99%)
33	ORF42, 93	323/323(100%)
34	ORF43, 93	145/150(96%)
35	ORF44, 93	150/153(98%)
36	ORF45, 93	73/78(93%)
37	ORF46, 93	56/57(98%)
38	ORF40, 107	51/51(100%)
39	ORF46, 107	15/36(41%)
40	-	-
41	ORF43, 107	111/183(60%)
42	ORF36, 128	162/168(96%)
43	ORF37, 128	84/100(84%)
44	ORF46, 107	214/235(91%)
45	ORF40, 53	132/132(100%)
46	ORF41, 53	168/172(97%)

*: in gray are proteins with higher similarity with proteins of other uruguayan phages

TABLE S6: Phage 128 proteins comparition against other uruguayan phages*

ORF	BLAST aganst Uruguayan phages	% Identity (aa)
1	ORF1, 53	143/151(95%)
2	ORF2, 73	605/623(97%)
3	ORF3,& 73	59/59(100%)
4	ORF4, 73	386/386(100%)
5	ORF6, 53	222/222(100%)
6	ORF6, 107; ORF7, 53	393/397(99%)
7	ORF7, 73 & 107; ORF8, 53	104/104(100%)
8	ORF8, 73, 107	116/116(100%)
9	ORF9, 73; ORF10, 53	140/140(100%)
10	ORF10, 73; ORF11, 53	121/123(98%)
11	ORF11, 73; ORF12, 53	197/203(97%)
12	ORF12, 107	114/117(97%)
13	ORF13, 107	1362/1624(84%)
14	ORF14, 73	421/527(80%)
15	ORF15, 107	*2
16	ORF20, 93	566/687(82%)
17	ORF21, 93	116/132(88%)
18	ORF22, 93	38/48(79%)
19	ORF19, 73	101/107(94%)
20	ORF24, 93	69/80(86%)
21	ORF22, 53	251/281(89%)
22	ORF24, 73	60/67(90%)
23	ORF27, 107	230/230(100%)
24	ORF27, 73	68/69(99%)
25	ORF28, 53	40/44(91%)
26	-	-
27	ORF30, 53	214/222(96%)
28	ORF31, 53	415/443(94%)
29	ORF32, 53	147/151(97%)
30	ORF33, 53	264/271(97%)
31	ORF34, 53	481/502(96%)
32	ORF35, 53	101/107(94%)
33	ORF36, 53	71/79(90%)
34	ORF46, 93	49/57(86%)
35	ORF47, 93	45/50(90%)
36	ORF42, 73	152/168(90%)
37	ORF43, 73	75/100(75%)
38	ORF39, 53	198/235(84%)
39	ORF46, 107	126/132(95%)
40	ORF47, 107	163/172(95%)

*: in gray are proteins with higher similarity with proteins of other uruguayan phages

*2: 1-501:471/501(94%); 679-1059: 301/381(79%)

Table S7. Sequences of the spacers in the CRISPR1 of *S. thermophilus* UY01

Spacer #	Sequence 5´ - 3´	Best matches and number of spacers	ID
17	AGCAAATTGATGCCATTGTTTCTCTCCTCC	*S. thermophilus* prophage TP-J34 *orf14*	30/30
		S. thermophilus phage 5093 *orf5*	30/30
16	ATGATGATGAAGTATCGTCATCTACTAAC	*S. thermophilus* strain LMD-9/UY03 CRISPR1 #16	29/29
15	CTTCACCTCAAATCTTAGAGCTGGACTAAA	*S. thermophilus* strain LMD-9/UY03 CRISPR1 #15	30/30
		S. thermophilus phage 7201 *orf39*	30/30
14	ATGTCTGAAAAATAACCGACCATCATTACT	*S. thermophilus* strain LMD-9/UY03 CRISPR1 #14	30/30
	ATGTCTGAAAAA**T**AACCGACCATCATTACT	*S. thermophilus* prophage TP-778L *orf669*	29/30
13	GAAGCTCATCATGTTAAGGCTAAAACCTAT	*S. thermophilus* strain LMD-9/UY03 CRISPR1 #13	30/30
		S. thermophilus phage 128 *orf28*	30/30
12	TAGTCTAAATAGATTTCTTGCACCATTGTA	*S. thermophilus* strain LMD-9/UY03 CRISPR1 #12	30/30
11	ATTCGTGAAAAAATATCGTGAAATAGGCAA	*S. thermophilus* strain LMD-9/UY03 CRISPR1 #11	30/30
10	TCTAGGCTCATCTAAAGATAAATCAGTAGC	*S. thermophilus* strain LMD-9/SMQ-301/UY03 CRISPR1 #10	30/30
9	TAAAAACATGGGGCGGCGGTAATAGTGTAAG	*S. thermophilus* strain LMD-9/SMQ-301/UY03 CRISPR1 #9	31/31
8	ACAACCAGCAAAGAGAGCGCCGACAACATT	*S. thermophilus* strain LMD-9/SMQ-301/UY03 CRISPR1 #8	30/30
7	TATAACACAGGTTTAGAGGATGTTATACTT	*S. thermophilus* strain LMD-9/SMQ-301/UY03 CRISPR1 #7	30/30
6	CTAGAAGCTCAAGCGGTAAAAGTTGATGGCG	*S. thermophilus* strain LMD-9/SMQ-301/UY03 CRISPR1 #6	31/31
5	CTTTGAGGGCAAGCCCTCGCCGTTCCATTT	*S. thermophilus* strain LMD-9/SMQ-301/UY03 CRISPR1 #5	30/30
4	AACTACCAAGCAAATCAGCAATCAATAAGT	*S. thermophilus* strain LMD-9/SMQ-301/UY03 CRISPR1 #4	30/30
3	CTATAAGTGACAATCAGCGTAGGGAATACG	*S. thermophilus* strain LMD-9/SMQ-301/UY03 CRISPR1 #3	30/30
2	ATCAGTGCGGTATATTTACCCTAGACGCTA	*S. thermophilus* strain LMD-9/SMQ-301/UY03 CRISPR1 #2	30/30
1	AACAGTTACTATTAATCACGATTCCAACGG	*S. thermophilus* strain LMD-9/SMQ-301/UY03 CRISPR1 #1	30/30
	AACAGTTAC**T**ATTAATCACGATTC**CA**ACGG	*S. thermophilus* phage 53 *orf17*	27/30

Table S8. Sequences of the spacers in the CRISPR1 of *S. thermophilus* UY02

Spacer #	Sequence 5´ – 3´	Best matches and number of spacers	ID
23	GTGAAATGCTTTTTCTAATTCATGTGGTCT	No hit	
22	TTAAGTGGTATTATTATATTATCGAAGAAG	*S. thermophilus* prophage 20617 intergenic	30/30
		S. thermophilus phage 5093 intergenic	30/30
		S. thermophilus phage 858 intergenic	30/30
		S. thermophilus phage 2972 intergenic	30/30
		S. thermophilus phage Sfi11 intergenic	30/30
21	GCAACAGT**A**AAACGTTGCAAACGAAAACTT	*S. thermophilus* phage O1205 *orf24*	29/30
20	TTCCCGGCGTATATACTGGCTCGATTGTTT	No hit	
19	CAATAGTTACCCGAGTACCATCTTCAAGCA	No hit	
18	AACACAGCAAGACAAGAGGATGATGCTATG	*S. thermophilus* strain DGCC7710 CRISPR1 #20	30/30
		S. thermophilus prophage 20617 gene hel	30/30
		S. thermophilus prophage TP-J34 *orf11*	30/30
		S. thermophilus phage 5093 *orf2*	30/30
17	AGAAGTCACTCGTGAGAAACACTACTCAAA	*S. thermophilus* phage 7201 *orf29*	30/30
16	CTTTTTTGGCAATCCAACCTGAGAGCCAAG	No hit	
15	TGCAAACAAAACAGTGCGATCGCTTGCAAG	*S. thermophilus* phage 7201 *orf18*	30/30
14	AATTAAGGGCATAGAAAGGGAGACAACATG	*S. thermophilus* strain DGCC7770 CRISPR1 #17	30/30
		S. thermophilus prophage 20617 gene rec	30/30
13	CGATATTTAAAATCATTTTCATAACTTCAT	*S. thermophilus* strain DGCC7710 CRISPR1 #16	30/30
12	GCAGTATCAGCAAGCAAGCTGTTAGTTACT	*S. thermophilus* strain DGCC7710 CRISPR1 #15	30/30
	GCAG**TA**TCAGCAAGCAAGCTGTTAGT**T**ACT	*S. thermophilus* phage 128 *orf6*	27/30
11	ATAAACTATGAAATTTTATAATTTTTAAGA	*S. thermophilus* strain DGCC7710 CRISPR1 #14	30/30
		S. thermophilus phage 7201 *orf8*	30/30
		S. thermophilus prophage 20617 gene e10	30/30
10	TGGAAACTAAGAAATGCAATAGAGTGGAAG	*S. thermophilus* strain UY02 CRISPR1 #5	30/30
	TGGAAAC**T**AAGAAATGCAATAGA**G**TGGAAG	*S. thermophilus* phage Abc2 *orf45*	28/30
	TGGAAAC**T**AAGAAATGCAATAGA**G**TGGAAG	*S. thermophilus* phage 858 *orf46*	28/30
	TGGAAAC**T**AAGAAATGCAATAGA**G**TGGAAG	*S. thermophilus* phage 2972 *orf44*	28/30
	TGGAAAC**T**AAGAAATGCAATAGA**G**TGGAAG	*S. thermophilus* phage Sfi19 *orf235*	28/30
9	AAATCTCGTAGTTAGTACAGTAGGTTTCAA	*S. thermophilus* phage Sfi19 *orf161*	30/30
8	ATAACTGAAGGATAGGAGCTTGTAAAGTCT	*S. thermophilus* strain DGCC7710 CRISPR1 #6	30/30
7	TAATGCTACATCTCAAAGGATGATCCCAGA	*S. thermophilus* strain DGCC7710 CRISPR1 #5	30/30
		S. thermophilus phage Sfi21 *orf670*	30/30
		S. thermophilus phage Sfi19 *orf670*	30/30
6	GAAAAAGCATCCATGATAGTGCTTAGACCT	*S. thermophilus* strains DGCC7796 CRISPR1 #9	30/30
5	TGGAAACTAAGAAATGCAATAGAGTGGAAG	*S. thermophilus* strain UY02 CRISPR1 #10	30/30
	TGGAAAC**T**AAGAAATGCAATAGA**G**TGGAAG	*S. thermophilus* phage Abc2 *orf45*	28/30
	TGGAAAC**T**AAGAAATGCAATAGA**G**TGGAAG	*S. thermophilus* phage 858 *orf46*	28/30
	TGGAAAC**T**AAGAAATGCAATAGA**G**TGGAAG	*S. thermophilus* phage 2972 *orf44*	28/30
	TGGAAAC**T**AAGAAATGCAATAGA**G**TGGAAG	*S. thermophilus* phage Sfi19 *orf235*	28/30
4	AAGTAGTTGATGACCTCTACAATGGTTTAT	*S. thermophilus* strain DGCC7710 CRISPR1 #4	30/30
3	ACCTAGAAGCATTTGAGCGTATATTGATTG	*S. thermophilus* strain DGCC7710 CRISPR1 #3	30/30
2	AATTTTGCCCCTTCTTTGCCCCTTGACTAG	*S. thermophilus* strain DGCC7710 CRISPR1 #2	30/30
1	ACCATTAGCAATCATTTGTGCCCATTGAGT	*S. thermophilus* strain DGCC7710 CRISPR1 #1	30/30

Table S9. Sequences of the spacers in the CRISPR1 of *S. thermophilus* UY03

Spacer #	Sequence 5´ - 3´	Best matches and number of spacers	ID
16	ATGATGATGAAGTATCGTCATCTACTAAC	*S. thermophilus* strain LMD-9/UY01 CRISPR1 #16	29/29
15	CTTCACCTCAAATCTTAGAGCTGGACTAAA	*S. thermophilus* strain LMD-9/UY01 CRISPR1 #15	30/30
		S. thermophilus phage 7201 *orf39*	30/30
14	ATGTCTGAAAAATAACCGACCATCATTACT	*S. thermophilus* strain LMD-9/UY01 CRISPR1 #14	30/30
	ATGTCTGAAAAA**T**AACCGACCATCATTACT	*S. thermophilus* prophage TP-778L *orf669*	29/30
13	GAAGCTCATCATGTTAAGGCTAAAACCTAT	*S. thermophilus* strain LMD-9/UY01 CRISPR1 #13	30/30
		S. thermophilus phage 128 *orf28*	30/30
12	TAGTCTAAATAGATTTCTTGCACCATTGTA	*S. thermophilus* strain LMD-9/UY01 CRISPR1 #12	30/30
11	ATTCGTGAAAAAATATCGTGAAATAGGCAA	*S. thermophilus* strain LMD-9/UY01 CRISPR1 #11	30/30
10	TCTAGGCTCATCTAAAGATAAATCAGTAGC	*S. thermophilus* strain LMD-9/SMQ-301/UY01 CRISPR1 #10	30/30
9	TAAAAACATGGGGCGGCGGTAATAGTGTAAG	*S. thermophilus* strain LMD-9/SMQ-301/UY01 CRISPR1 #9	31/31
8	ACAACCAGCAAAGAGAGCGCCGACAACATT	*S. thermophilus* strain LMD-9/SMQ-301/UY01 CRISPR1 #8	30/30
7	TATAACACAGGTTTAGAGGATGTTATACTT	*S. thermophilus* strain LMD-9/SMQ-301/UY01 CRISPR1 #7	30/30
6	CTAGAAGCTCAAGCGGTAAAAGTTGATGGCG	*S. thermophilus* strain LMD-9/SMQ-301/UY01 CRISPR1 #6	31/31
5	CTTTGAGGGCAAGCCCTCGCCGTTCCATTT	*S. thermophilus* strain LMD-9/SMQ-301/UY01 CRISPR1 #5	30/30
4	AACTACCAAGCAAATCAGCAATCAATAAGT	*S. thermophilus* strain LMD-9/SMQ-301/UY01 CRISPR1 #4	30/30
3	CTATAAGTGACAATCAGCGTAGGGAATACG	*S. thermophilus* strain LMD-9/SMQ-301/UY01 CRISPR1 #3	30/30
2	ATCAGTGCGGTATATTTACCCTAGACGCTA	*S. thermophilus* strain LMD-9/SMQ-301/UY01 CRISPR1 #2	30/30
1	AACAGTTACTATTAATCACGATTCCAACGG	*S. thermophilus* strain LMD-9/SMQ-301/UY01 CRISPR1 #1	30/30
	AACAGTTACTATTAATCACGATTC**CA**ACGG	*S. thermophilus* phage 73 *orf16*	28/30

Table S10. Sequences of the spacers in the CRISPR3 of *S. thermophilus* UY01

Spacer #	Sequence 5´ - 3´	Best matches and number of spacers	ID
9	TATGCAAGTAAAGGAATATGCTTTATATAA	*S. thermophilus* phage 128 *orf33*	30/30
8	GGTGAAAAAGGTTCACTGTACGAGTACTTA	*S. thermophilus* strain LMD-9 CRISPR 3 #8	30/30
7	TCAATGAGTGGTATCCAAGACGAAAACTTA	*S. thermophilus* strain LMD-9 CRISPR 3 #7	30/30
6	CCTTGTCGTGGCTCTCCATACGCCCATATA	*S. thermophilus* strain LMD-9 CRISPR 3 #6	30/30
		S. thermophilus plasmid pND103 intergenic	30/30
5	TGTTTGGGAAACCGCAGTAGCCATGATTAA	*S. thermophilus* strain LMD-9 CRISPR 3 #5	30/30
		S. thermophilus strain SMQ-301/UY03 CRISPR3 #13	30/30
		S. thermophilus phage 7201 *orf33*	30/30
		S. thermophilus phage 128 *orf13*	30/30
4	ACAGAGTACAATATTGTCCTCATTGGAGACAC	*S. thermophilus* strain LMD-9 CRISPR 3 #4	32/32
		S. thermophilus strain SMQ-301/UY03 CRISPR3 #12	30/30
		S. thermophilus prophage TP-J34 *orf11*	32/32
3	CTCATATTCGTTAGTTGCTTTTGTCATAAA	*S. thermophilus* strain LMD-9 CRISPR3 #3	30/30
		S. thermophilus strain SMQ-301/UY03 CRISPR3 #5	30/30
		S. thermophilus phage Sfi19 *orf1626*	30/30
		S. thermophilus phage 128 *orf13*	30/30
	CTCATATTCGTT**A**GT**T**GCTTTTGT**C**ATAAA	*S. thermophilus* phage 53 *orf14*	27/30
2	AGAACTTTATCAAGATAAAACTACTTTAAA	*S. thermophilus* strain LMD-9/SMQ-301/UY03 CRISPR3 #2	30/30
1	ATAGTATTAATTTCATTGAAAAATAATTGT	*S. thermophilus* strain LMD-9/SMQ-301/UY03 CRISPR3 #1	30/30

Table S11. Sequences of the spacers in the CRISPR3 of *S. thermophilus* UY03

Spacer #	Sequence 5´ - 3´	Best matches and number of spacers	ID
16	GAATTTGCTTGAAGGGACTAAAGACTTTAG	*S. thermophilus* phage MD2 host recognition gene	30/30
	GAATTTGCTTGAAGGGACTAAA**G**A**C**TTTAG	*S. thermophilus* phage 73 *orf15*	28/30
15	AATTGTAAAATCGTGCTACGGGCGTTTTAT	*S. thermophilus* plasmid pK1002C2	30/30
		S. thermophilus plasmid pK2007C6	30/30
		S. thermophilus strain LMD-9 plasmid 2	30/30
14	TCTGACGGTTAGATATGATTTTACTGGTAA	*S. thermophilus* strain SMQ-301 CRISPR3 #14	30/30
	TCTGACGGTTAGATAT**G**ATTTTACTGGTAA	*S. thermophilus* phage 858 *orf22*	29/30
	TCTGACGGTTAGATAT**G**ATTTTACTGGTAA	*S. thermophilus* phage 2972 *orf21*	29/30
13	TGTTTGGGAAACCGCAGTAGCCATGATTAA	*S. thermophilus* strain SMQ-301 CRISPR3 #13	30/30
		S. thermophilus strain LMD-9/UY01 CRISPR3 #5	30/30
		S. thermophilus phage 7201 *orf33*	30/30
		S. thermophilus phage 128 *orf13*	30/30
12	ACAGAGTACAATATTGTCCTCATTGGAGACAC	*S. thermophilus* strain SMQ-301 CRISPR3 #12	32/32
		S. thermophilus strain LMD-9/UY01 CRISPR3 #4	30/30
11	TGATGGACGAGACGGTATTCCAGGAAAACC	*S. thermophilus* strain SMQ-301 CRISPR3 #11	30/30
10	ATTGGAAAAAGGCGTTTTTACTAATGAGTA	*S. thermophilus strain UY03 CRISPR3 #8*	30/30
		S. thermophilus strain SMQ-301 CRISPR3 #8 and #10	30/30
9	ATACTTACGATGGCGAAGATTACAACTATAG	*S. thermophilus* strain UY03 CRISPR3 #7	31/31
		S. thermophilus strain SMQ-301 CRISPR3 #7 and #9	31/31
8	ATTGGAAAAAGGCGTTTTTACTAATGAGTA	*S. thermophilus* strain UY03 CRISPR3 #10	30/30
		S. thermophilus strain SMQ-301 CRISPR3 #8 and #10	30/30
7	ATACTTACGATGGCGAAGATTACAACTATAG	*S. thermophilus* strain UY03 CRISPR3 #9	31/31
		S. thermophilus strain SMQ-301 CRISPR3 #7 and #9	31/31
6	TATTGAAACGAGCGTGCCTTTTAAGCCATC	*S. thermophilus* strain SMQ-301 CRISPR3 #6	30/30
5	CTCATATTCGTTAGTTGCTTTTGTCATAAA	*S. thermophilus* strain SMQ-301 CRISPR3 #5	30/30
		S. thermophilus strain LMD-9/UY01 CRISPR3 #3	30/30
		S. thermophilus phage Sfi19 *orf1626*	30/30
		S. thermophilus phage 128 *orf13*	30/30
	CTCATATTCGTT**A**GT**T**GCTTTTGT**C**ATAAA	*S. thermophilus* phage 73 *orf13*	27/30
4	TGAATCTTCTAACTTTAACTCAGTTGTTAC	*S. thermophilus* strain SMQ-301 CRISPR3 #4	30/30
		S. thermophilus phage 858 *orf40*	30/30
3	AATAATAAAAGTGATACAAGCTCAAGGCAA	*S. thermophilus* strain SMQ-301 CRISPR3 #3	30/30
2	AGAACTTTATCAAGATAAAACTACTTTAAA	*S. thermophilus* strain LMD-9/SMQ-301/UY01 CRISPR3 #2	30/30
1	ATAGTATTAATTTCATTGAAAAATAATTGT	*S. thermophilus* strain LMD-9/SMQ-301/UY01 CRISPR3 #1	30/30
